# Antrodia camphorata polysaccharide improves inflammatory response in liver injury via the ROS/TLR4/NF‐κB signal

**DOI:** 10.1111/jcmm.17283

**Published:** 2022-03-29

**Authors:** Yi Yang, Chenyang Han, Yongjia Sheng, Jin Wang, Wenyan Li, Xiaohong Zhou, Shuiliang Ruan

**Affiliations:** ^1^ 569220 Department of Pharmacy The Second Affiliated Hospital of Jiaxing University Jiaxing China; ^2^ 569220 Department of Digestive The Second Affiliated Hospital of Jiaxing University Jiaxing China

**Keywords:** Antrodia Camphorata Polysaccharide, Kupffer cells, liver injury, NLRP3, Nrf2 signal

## Abstract

Antrodia Camphorata Polysaccharide (ACP) refers to a kind of polysaccharide extracted from the natural porous fungus Antrodia camphorata. This study investigated the mechanism of action of ACP in protecting the liver. The results showed that ACP suppressed the LPS‐induced KC cell activation, reduced the expression of inflammatory factors, increased the SOD level and suppressed ROS expression. In addition, N‐acetylcysteine (NAC) was adopted for pre‐treatment to suppress ROS. The results indicated that NAC synergistically exerted its effect with ACP, suggesting that ACP played its role through suppressing ROS. Further detection revealed that ACP activated the Nrf2 signal. It was discovered in the mouse model that, ACP effectively improved liver injury in mice, decreased ALT and AST levels, and suppressed the expression of inflammatory factors. This study suggests that ACP can exert its effect against oxidative stress via the Nrf2‐ARE signalling, which further improves the production of ROS and the activation of TLR4‐NF‐κB signalling, and protects the liver against liver injury.

## 1 BACKGROUND

Liver injury is the consequence of hepatic pathological changes induced by various factors, and its commonly seen types in clinic include virus infection, drug injury and alcohol injury.^1^ In liver injury, the liver manifests as tissue necrosis, cholestasis, fibrosis, liver cirrhosis and liver cancer.^2^ Acute liver injury is one of the common liver injury types, which involves complicated pathological factors.^3^ Oxidative stress (OS) refers to a process of inflammation and inflammatory cell infiltration resulting from the imbalance of active molecules such as reactive oxygen species (ROS) when the body is subject to stimuli.^4^, ^5^ It is discovered that, the excessive expression of ROS in liver injury can activate NF‐κB via the Toll‐like receptor 4(TLR4)signal, leading to the persistent and strong inflammatory response.^6^ As a result, a large number of inflammatory factors are released, which can induce liver injury.^7^ In liver injury research, D‐galactosamine/lipopolysaccharide (D‐GalN/LPS) is a classical chemical substance that results in liver injury. D‐GalN/LPS can be activated by the cytochrome P450 of hepatocytes to produce trichloromethyl free radicals and peroxy radicals, thus directly inducing peroxidation injuries of hepatocyte DNA, proteins and lipids.^8^ Additionally, D‐GalN/LPS also induce inflammatory response of hepatocytes, thereby further promoting liver injury. At present, drugs such as silymarin and bifendate are commonly used in the treatment of liver injury. As for natural drugs, baicalin and Chinese Angelica extract have also been verified to achieve favourable therapeutic effect on liver injury.^9^

Antrodia Camphorata (AC) is a Polypores family fungus unique to Taiwan, which mainly grows in Pingtung, Taiwan. Its taxonomy is similar to the well‐known Ganoderma lucidum and Trametes versicolor in Mainland China.^10^ Through exploring the composition of AC, we discovered that the contents of Antrodia Camphorata Polysaccharide (ACP) and triterpenoids in AC are significantly higher than those in fungi of the same type, which are of high development and research value.^11^ In local Taiwan, AC is mainly used to treat liver injury, alcoholic liver, non‐alcoholic fatty liver disease (NAFLD) and liver cancer, and can achieve favourable therapeutic efficacy.^12^ However, its exact material basis and pharmacodynamic effect have not been illustrated. Considering the high polysaccharide contents in AC, the current work aimed to illustrate the role and mechanism of ACP in resisting acute liver injury, aiming to provide a new thinking for the liver‐protection pharmacological action of AC.

## 2 MATERIALS AND METHODS

### 2.1 The role of ACP in Kupffer cell inflammatory response and the regulatory mechanism

The mouse Kupffer cells (KCs, Wuhan Procell Biotechnology Co., Ltd, Wuhan, China) were cultured with RMPI‐1640 complete medium. After confirming cell viability >90% by adopting trypan blue reagent, KCs were divided into DMSO, LPS and ACP groups. DMSO group was the control group, cells in LPS groups were treated with 0.5 mg/L LPS to induce inflammatory response, while cells in ACP groups (It is mainly composed of mannose (content 86.80%), and contains a small amount of rhamnose, arabinose, fucose, xylose and glucose) were pre‐treated with high (15 mg/L) and low (5 mg/L) concentrations of ACP for 6 h and then exposed to LPS to induce inflammatory response. The detection indexes were shown below.

#### 2.1.1 Flow cytometry

To be specific, KCs were inoculated into the 6‐well plates and grown to 80% before drug intervention. At 12 h after LPS induction, all cells were collected, which were washed with the pre‐chilled PBS, and centrifuged at 1500 *g* for 30 min. Thereafter, cells were stained after suspension with Binding Buffer, and incubated with 5 μl Annexin V‐FITC for 5 min in dark and then with 5 μl PI for another 5 min in dark in cell apoptosis detection kit (BD, Massachusetts, USA). After PBS washing, the cells were loaded for detection using the Annexin V‐FITC (+) PI (+) and Annexin V‐FITC (+) PI (−) apoptosis cytometers.

#### 2.1.2 Enzyme‐linked immunosorbent assay

The levels of secretory inflammatory factors IL‐1β, IL‐18 and TNF‐α in culture medium were detected. Briefly, after LPS induction for 12 h, the cell culture medium of KCs was collected and centrifuged to remove the suspension cells and cell debris. Next, the supernatants were collected for culture. In line with the enzyme‐linked immunosorbent assay (ELISA) kit (Nanjing Jiancheng Biological Engineering Research Institute, Nanjing, China) instructions, the inflammatory factor levels were detected by the standard curve method, and the results were expressed as pg/ml.

#### 2.1.3 Detection of SOD and MDA levels

After LPS induction for 12 h, the suspension and adhering KCs were collected, which were washed with pre‐chilled PBS, and lysed with cell lysis buffer on ice. Thereafter, cells were centrifuged at 4°C to collect the supernatants, and then SOD expression was detected using the WST kit (Nanjing Jiancheng Biological Engineering Research Institute, Nanjing, China), and the results were expressed as U/mg. While MDA expression was detected based on TAB kit (Nanjing Jiancheng Biological Engineering Research Institute, Nanjing, China), and the results were expressed as μmol/mg.

#### 2.1.4 Immunofluorescence staining

In brief, sterile slides were added into the 6‐well plates, inoculated with KCs and cultured to cell adherence. After LPS induction for 12 h, cells were fixed with 4% paraformaldehyde, permeabilized with 0.2% Triton X‐100, blocked with 2% BSA and incubated with CD68 goat anti‐mouse monoclonal antibody (dilution, 1:200; Abcam, Massachusetts, USA) at room temperature. Afterwards, cells were further incubated with fluorescence‐labelled IgG antibody (Abcam, Massachusetts, USA). At the same time, nuclei were stained with 0.5 μg/ml DAPI (Solarbio, Beijing, China). After washing twice with PBS, the slides were sealed and observed under the fluorescence microscope.

#### 2.1.5 ROS expression was detected by dihydroethidium

After LPS induction for 12 h, the KCs were collected and the cell density was adjusted at 10^6^/ml. Thereafter, the dihydroethidium (DHE) probe (Beyotime Biotechnology Co., Ltd, Shanghai, China) was utilized to detect ROS. Specifically, the DHE probe was diluted with serum‐free medium at 1:1000. Subsequently, 1 ml medium that contained DHE probe was added into each well to further incubate for 30 min. Thereafter, the medium was discarded, and cells were washed with serum‐free medium twice. The cell staining level was observed under the fluorescence microscope. Besides, the absorbance (OD) value was measured with the fluorescence spectrophotometer.

#### 2.1.6 Western blot

After LPS induction for 12 h, KCs were scraped with a sterile cell scraper and the suspension cells were collected. Afterwards, cells were lysed with 1.0 ml NP‐40 lysate (Beyotime Biotechnology Co., Ltd, Shanghai, China) on ice for 30 min, the protein solution was diluted with 5× loading buffer to 20 μl, and boiled for 8 min before SDS‐PAGE. Thereafter, the proteins were transferred onto PVDF membranes for 0.5–2 h. Then, the membranes were blocked with 5% skim milk for 2 h, incubated with anti‐TLR4 and anti‐p‐IкB monoclonal antibodies (diluted with TBST to 1:500) at 4°C overnight, and subsequently incubated with HRP‐IgG (Abcam, USA). Next, protein blots were detected by chemiluminescence and OD values were analysed using the Image Pro‐Plus 6.0 software. The results were expressed as the OD ratio of target protein to internal reference protein.

### 2.2 Effect of ACP on KC inflammatory response after NAC was used to suppress ROS

To verify that ACP regulated the activation of TLR4/NF‐κB signal via ROS, KCs were divided into DMSO, LPS, NAC, and NAC + ACP groups. Cells in NAC group were pre‐treated with 10 μM NAC for 6 h, while those in NAC + ACP group were pre‐treated with 15 mg/L ACP and 10 μM NAC for 6 h, and then exposed to 0.5 mg/L LPS to induce inflammatory response. In line with the above‐mentioned experimental operation, cell apoptosis was detected by flow cytometry, secretory inflammatory factors were detected by ELISA, SOD and MDA levels were measured using kit, ROS production was detected with DHE probe, and protein expression levels were measured by Western blot (WB) assay.

### 2.3 ACP activated Keap1‐Nrf2 signal to exert the anti‐ROS effect

To explore the mechanism by which ACP suppressed ROS, KCs were divided into LPS, BCI and ACP groups. Cells in BCI group were pre‐treated with 5 μM Nrf2 agonist (E/Z)‐BCI to activate the Nrf2 signal, while cells in ACP group were intervened with 15 mg/L ACP and then treated with 0.5 mg/L LPS to induce inflammatory response. The detection indexes were shown below.

1. CCK‐8: In brief, KCs were inoculated into the 96‐well plates. Three duplicate wells were set for each treatment, and blank medium was set as control. Cell viability was detected at 0, 3, 6 and 12 h after LPS intervention. Afterwards, 100 μl medium was replaced in each well. Then, 10 μl CCK‐8 solution (Beyotime Biotechnology Co., Ltd, Shanghai, China) was added to incubate for 2 h, followed by measurement of OD value at 450 nm.
2. ROS levels were detected by DHE probe according to the above‐mentioned description.
3. Nrf2 expression was measured by immunofluorescence staining in line with the above description, and Nrf2 monoclonal antibody (Abcam, Massachusetts, USA) was diluted with TBST at 1:300.
4. Nrf2 and Keap1 expression was detected by WB assay according to the above description, and monoclonal antibody (Abcam, Massachusetts, USA) was diluted with TBST at 1:500.

### 2.4 Intervention of ACP on mice with acute liver injury

The SPF grade C57BL/6 mice were randomly divided into Control group, D‐GalN/LPS (D/L) group and ACP group. Mice in ACP group were given gavage of ACP (5 mg/kg and 15 mg/kg) once daily for 7 consecutive days. Mice in Con group and D/L group were given gavage of normal saline at the same volume. At 24 h after the final administration, mice in D/L group and ACP group were given intraperitoneal injection of 1000 mg/kg D‐GalN (Sigma, Massachusetts, USA) and 10 μg/kg LPS (Sigma, Massachusetts, USA) to construct the acute liver injury model.

#### 2.4.1 H&E staining

At 72 h after LPS/D‐GalN injection, mice were sacrificed by carbon dioxide suffocation to dissect the liver tissues. After paraffin embedding and preparation into the 4‐μm consecutive sections, all sections were stained as follows in succession, xylene deparaffinage, gradient dehydration with 100%, 95% and 80% ethanol, washing with tap water for 2 min, and haematoxylin staining for 3 min. After washing with tap water for 2 min, the sections were treated with 1% hydrochloric acid alcohol for 2 s. After washing with tap water for 2 min again, the sections were processed with 1% ammonium hydroxide for 20 s and 0.5% eosin alcohol for 10 s, followed by gradient alcohol dehydration, xylene transparency and neutral gum mounting. Finally, the liver histopathological changes were observed under the light microscope.

#### 2.4.2 AST and ALT expression in peripheral blood of mice was detected by kits

At 72 h after LPS/D‐GalN intervention, the tail venous blood was collected from mice, and centrifuged to harvest the supernatants. Thereafter, ALT and AST were detected using the ultraviolet colourimetry (Nanjing Jiancheng Biological Engineering Research Institute, Nanjing, China) in line with kit instructions. The results were expressed as U/L.

#### 2.4.3 ELISA

Inflammatory factors IL‐1β, IL‐18 and TNF‐α were detected using the ELISA kit (Nanjing Jiancheng Biological Engineering Research Institute, Nanjing, China). Briefly, peripheral blood was collected and centrifuged to obtain the serum for detection, whereas liver tissues were cut into pieces using the sterile surgical scissors, grinded with liquid nitrogen and lysed with 1.0 ml RIPA lysate on ice for 30 min. The supernatants were collected for protein quantification in line with kit instructions. The results were expressed as pg/ml.

#### 2.4.4 Immunohistochemistry

The paraffin‐embedded liver tissue was prepared into 4‐μm consecutive sections, followed by xylene deparaffinage, gradient ethanol immersion (100%–95%–85%), and microwave heating at 98°C for antigen retrieval. Thereafter, sections were incubated with 3% hydrogen peroxide at room temperature for 10 min to eliminate the endogenous peroxidase. Afterwards, sections were blocked with 2% bovine serum albumin (BSA) at 37°C for 30 min, followed by standing to allow for non‐specific binding of antigen to antibody (the primary antibody was NLRP3 diluted at 1:250, Abcam, USA). Next, each section was incubated with peroxidase‐labelled streptomycin (Abcam, USA) for 15 min. Subsequently, the freshly prepared DAB solution (DAKO, Denmark) was added for colour development. After sufficient washing with tap water, sections were counter‐stained with haematoxylin and sealed. All sections were photographed using the Olympus‐BX51 upright microscope equipped with the Olympus‐DP72 image collection system and CRi Nauance multi‐spectral imaging system (Cambridge Research & Instrumentation, USA).

#### 2.4.5 Expression of TLR4, p‐IкB, Nrf2 and Keap1 was detected by WB assay

The relative protein expression levels were detected according to the above‐mentioned detection methods.

### 2.5 Statistical analysis

The SPSS19.0 software was employed for performing the statistical analysis. Measurement data were expressed as mean ± standard deviation ($\bar{x}$ ± s), comparison amongst multiple groups was conducted by one‐way ANOVA, while comparison between two groups was carried out by SNK test. *p* < 0.05 indicated that the results were statistically significant.

## 3 RESULTS

### 3.1 Effect of ACP on the activation of KCs

**FIGURE 1:**
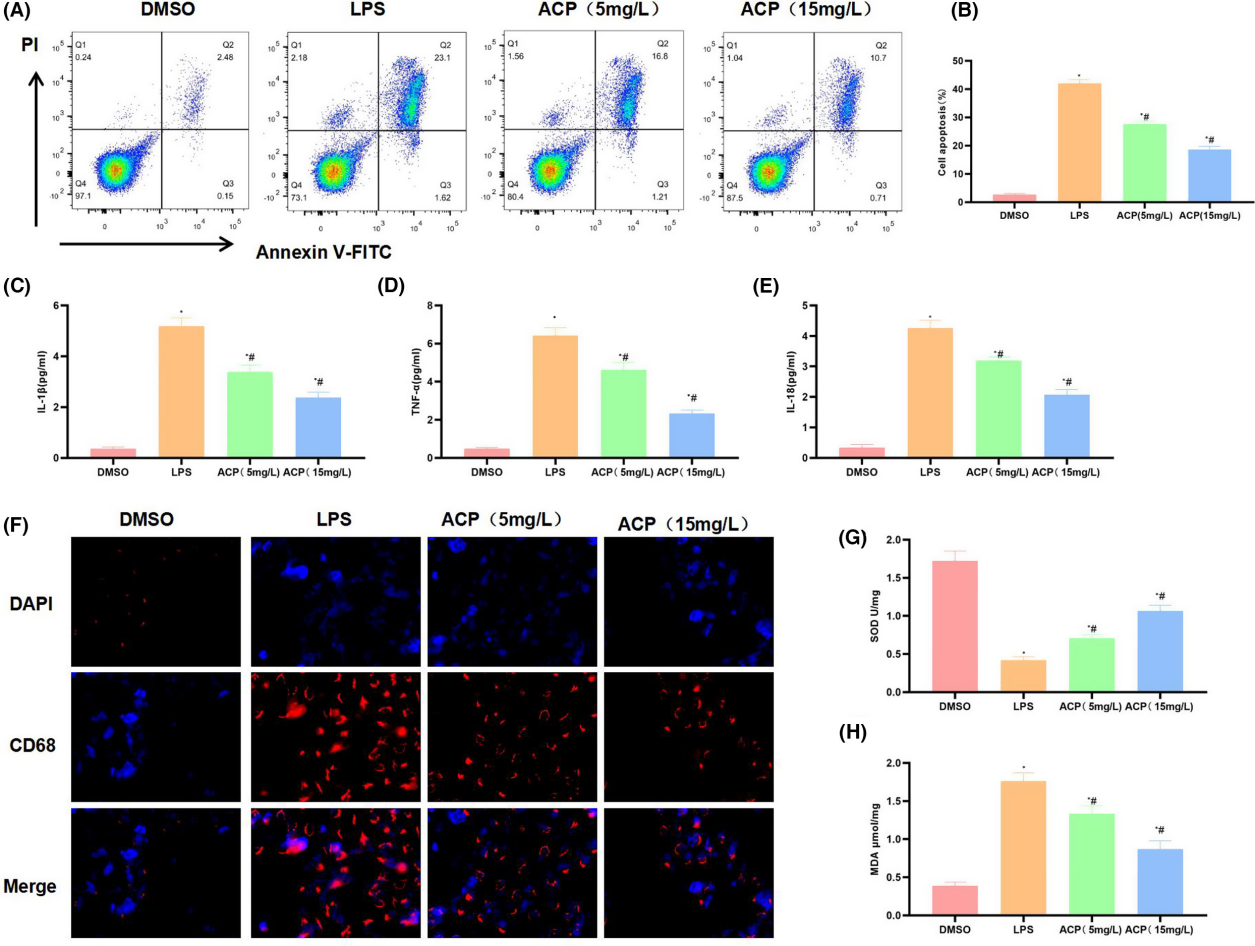
Effect of ACP on the activation of KCs (*n* = 3). (A, B) Cell apoptosis detection results. LPS promoted the inflammatory injury of KCs and increased the cell apoptosis rate, while ACP suppressed cell apoptosis in a dose‐dependent manner, and high‐dose ACP generated more obvious effect. ^*^ *p* < 0.05, compared with DMSO; ^#^ *p* < 0.05, compared with LPS. (C–E) Expression and release of inflammatory factors. LPS promoted the expression and release of IL‐1β, IL‐18 and TNF‐α in KCs. ACP significantly suppressed such change and lowered the levels of inflammatory factors. ^*^ *p* < 0.05, compared with DMSO; ^#^ *p* < 0.05, compared with LPS. (F) CD68 expression detected by immunofluorescence staining (*n* = 3). LPS activated KCs and up‐regulated CD68 expression, while ACP suppressed KC activation and decreased CD68 expression. (G, H) SOD and MDA detection results ($\bar{x}$ ± s, *n* = 3). LPS down‐regulated SOD expression and increased MDA level, whereas ACP significantly increased SOD expression, decreased MDA level, and had antagonistic effect on LPS. ^*^ *p* < 0.05, compared with DMSO; ^#^ *p* < 0.05, compared with LPS

LPS induced KC injury and increased the cell apoptosis rate, while ACP significantly suppressed the effect of LPS and remarkably decreased the cell apoptosis level. High‐dose ACP had more prominent effect on suppressing apoptosis (Figure 1A–B). It was discovered in the detection of inflammatory factors that, LPS promoted the expression and release of IL‐1β, IL‐18 and TNF‐α, whereas ACP suppressed the release of inflammatory factors and lowered their levels (Figure 1C–E). As discovered from SOD and MDA detection, SOD expression in LPS group was down‐regulated, and MDA expression was up‐regulated, suggesting the presence of oxidative stress (OS) injury, while ACP dramatically increased SOD level and decreased MDA level (Figure 1G, H). Based on CD68 detection, LPS induced KC activation, up‐regulated CD68 expression, and ACP suppressed CD68 expression. Moreover, the results of immunofluorescence staining also suggested that ACP suppressed CD68 expression (Figure 1F).

**FIGURE 2:**
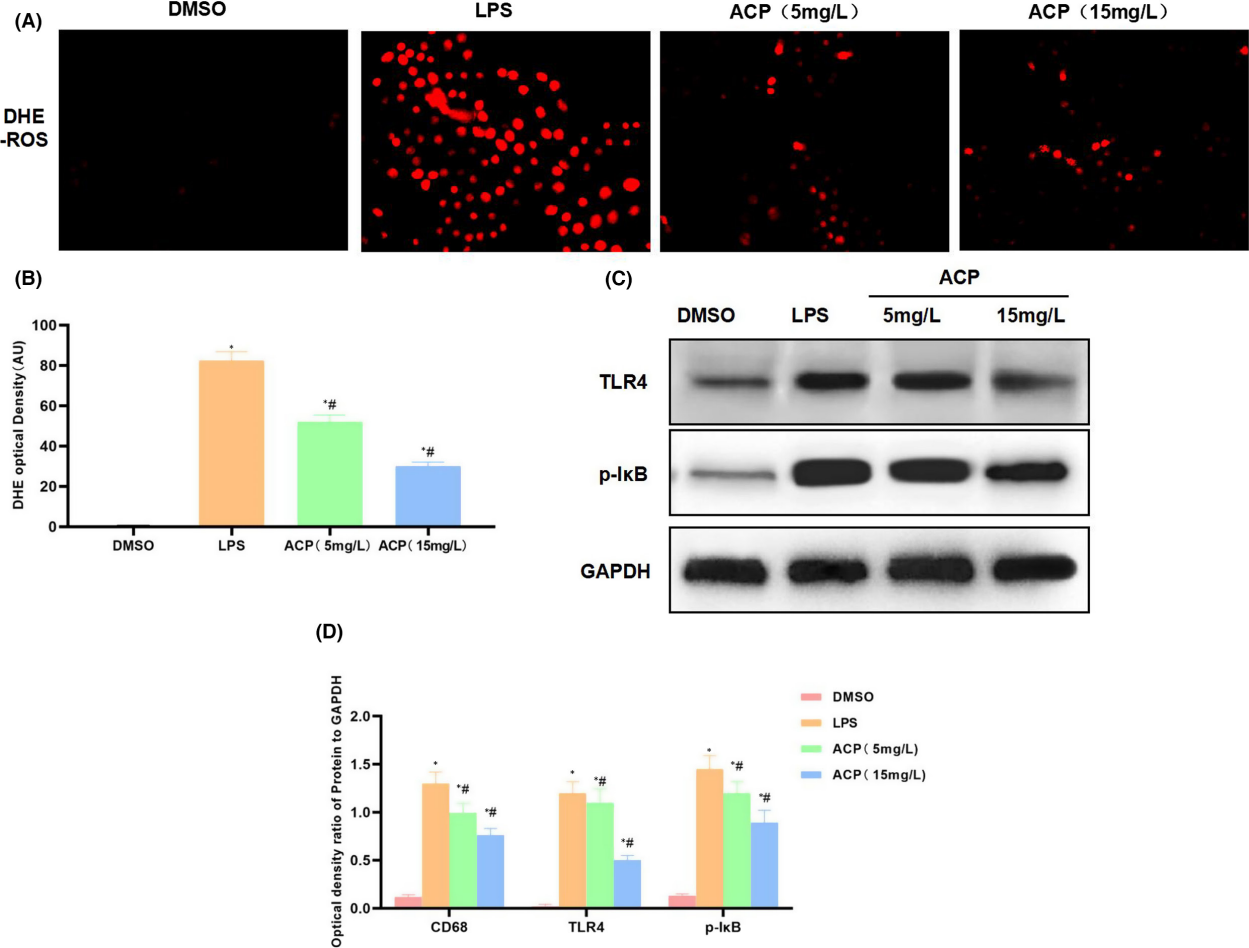
Effect of ACP on the ROS‐TLR4/NFκB signal in the activation of KCs (*n* = 3). (A, B) ROS expression detected by DHE probe. LPS activated ROS expression and significantly increased the fluorescence intensity, while ACP suppressed ROSDE level in a dose‐dependent manner, and the fluorescence intensity was significantly weakened compared with LPS. ^*^ *p* < 0.05, compared with DMSO; ^#^ *p* < 0.05, compared with LPS. (C, D) Protein expression detection results. LPS activated TLR4/NFκB signal, remarkably up‐regulated TLR4 and p‐IкB expression, while ACP suppressed TLR4 and p‐IкB expression. ^*^ *p* < 0.05, compared with DMSO; ^#^ *p* < 0.05, compared with LPS

Based on our mechanism research, when DHE probe was used to detect ROS, the ROS levels in DMSO were low, LPS substantially up‐regulated the ROS levels, with significantly higher fluorescence intensity than DMSO, while ACP dramatically suppressed ROS expression and further improved the ROS levels (Figure 2A, B). Protein detection results suggested that, LPS induced the expression of TLR4 and p‐IкB in the downstream ROS, and promoted the activation of ROS‐TLR4/NFκB signal. In the meanwhile, ACP suppressed the expression of TLR4 and p‐IкB and further down‐regulated the protein expression levels (Figure 2C, D).

### 3.2 ACP synergistically suppressed KC activation with NAC via ROS

**FIGURE 3:**
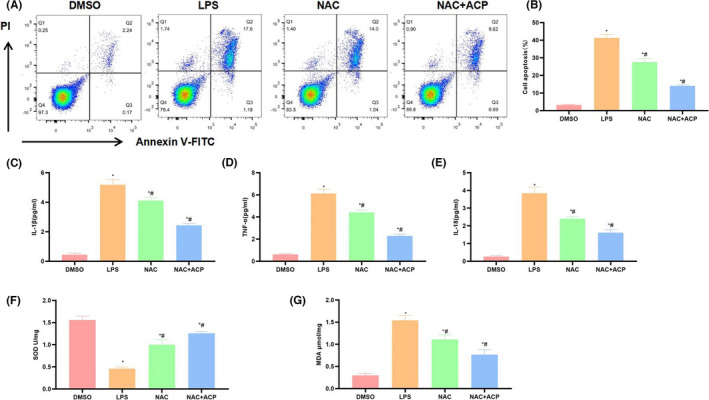
Effect of synergistic action of NAC with ACP in suppressing ROS on KC activation (*n* = 3). (A, B) Cell apoptosis detection results. NAC synergistically suppressed inflammatory injury of KCs with ACP and decreased the cell apoptosis rate. ^*^ *p* < 0.05, compared with DMSO; ^#^ *p* < 0.05, compared with LPS. (C–E) Inflammatory factor expression and release. LPS promoted the expression and release of IL‐1β, IL‐18 and TNF‐α in KCs, NAC synergistically decreased inflammatory factor expression with ACP. ^*^ *p* < 0.05, compared with DMSO; ^#^ *p* < 0.05, compared with LPS. (F, G) SOD and MDA detection results. LPS down‐regulated SOD expression and increased MDA level, whereas NAC + ACP significantly increased SOD level and decreased MDA expression. ^*^ *p* < 0.05, compared with DMSO; ^#^ *p* < 0.05, compared with LPS

**FIGURE 4:**
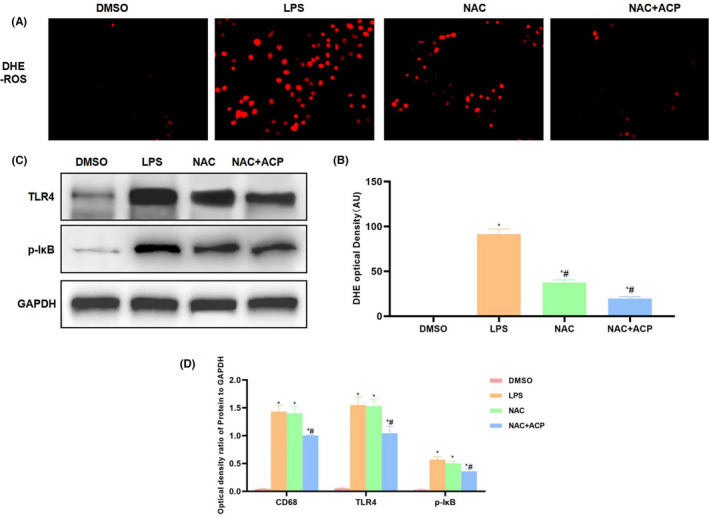
Effect of ACP on ROS‐TLR4/NFκB signal in KC activation (*n* = 3). (A, B) ROS expression detected by DHE ($\bar{x}$ ± s, *n* = 3). NAC suppressed ROS expression, NAC + ACP further inhibited ROS expression and exerted the synergistic effect. In comparison with LPS, the fluorescence intensity significantly decreased. ^*^ *p* < 0.05, compared with DMSO; ^#^ *p* < 0.05, compared with LPS. (C, D) Protein expression detection results ($\bar{x}$ ± s, *n* = 3). The expression of TLR4 and p‐IкB in LPS group was significantly up‐regulated, while NAC + ACP inhibited TLR4 and p‐IкB expression. ^*^ *p* < 0.05, compared with DMSO; ^#^ *p* < 0.05, compared with LPS

As discovered after NAC (the ROS inhibitor) pre‐treatment, ACP synergistically suppressed ROS production with NAC. According to cell apoptosis detection results, NAC + ACP group further down‐regulated the cell apoptosis level, and the difference was significant compared with NAC group (Figure 3A, B). Based on inflammatory factor detection results indicated that, NAC + ACP further down‐regulated the release of inflammatory factors (Figure 3C–E). In NAC + ACP group, SOD level further increased, while MDA level decreased (Figure 3F, G). DHE probe detection results indicated that, NAC suppressed ROS expression, while NAC + ACP further suppressed ROS expression, and the fluorescence intensity was significantly lower than NAC group (Figure 4A, B]. Besides, protein detection results demonstrated that, NAC exerted little influence on TLR4 signal, while NAC + ACP significantly suppressed the expression of TLR4 and p‐IкB (Figure 4C, D).

### 3.3 Mechanism of ACP in activating Nrf2 signal to suppress ROS expression

**FIGURE 5:**
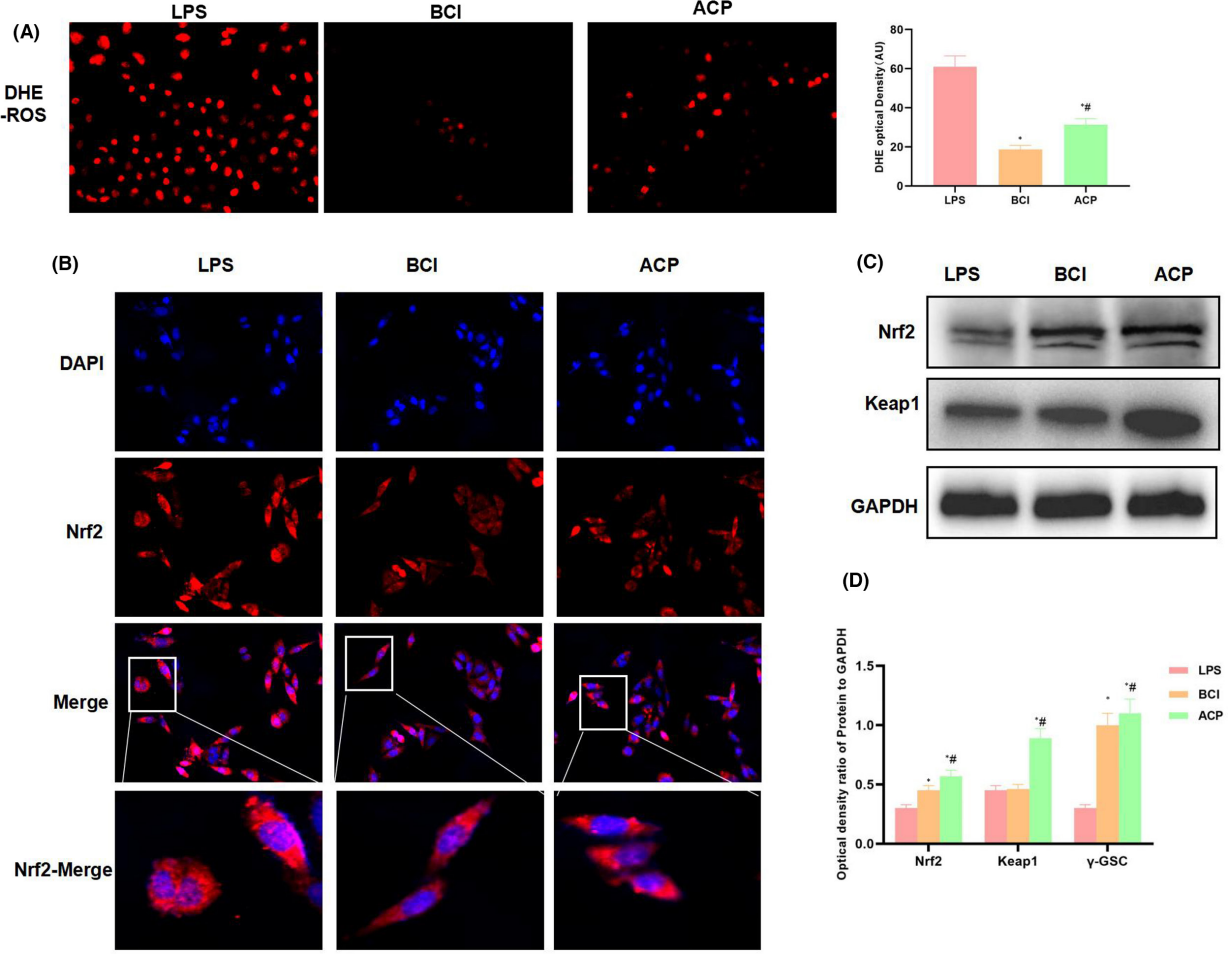
Mechanism of ACP in activating Nrf2 signal to exert the anti‐ROS effect (*n* = 3). (A) ROS expression detected by DHE. BCI activated Nrf2 to suppress ROS expression. Compared with LPS, the fluorescence intensity significantly decreased. ACP suppressed ROS levels. ^*^ *p* < 0.05, compared with LPS; ^#^ *p* < 0.05, compared with BCI. (B) Nrf2 expression detected by immunofluorescence staining. LPS activated the expression of Nrf2, while BCI and ACP further promoted NRf2 expression and improved its nuclear translocation. (C, D) Protein expression detection results. BCI activated Nrf2 signal, and up‐regulated the expression of Nrf2 and Keap1. Similarly, ACP also activated Nrf2 expression, and its effect was comparable to BCI. ^*^ *p* < 0.05, compared with LPS; ^#^ *p* < 0.05, compared with BCI

To explore the effect of ACP, we utilized the Nrf2 agonist BCI as the positive control. DHE detection results indicated that, BCI also suppressed ROS expression, similar to ACP (Figure 5A). In addition, immunofluorescence staining results also revealed that, LPS activated Nrf2 expression. Both BCI and ACP further promoted Nrf2 expression, and promoted its nuclear translocation and activation (Figure 5B). Similarly, protein detection results also suggested that both ACP and BCI promoted the expression of Nrf2 and Keap1, and exerted the anti‐oxidation effect (Figure 5C, D).

### 3.4 ACP improved the acute liver injury in mice via ROS

**FIGURE 6:**
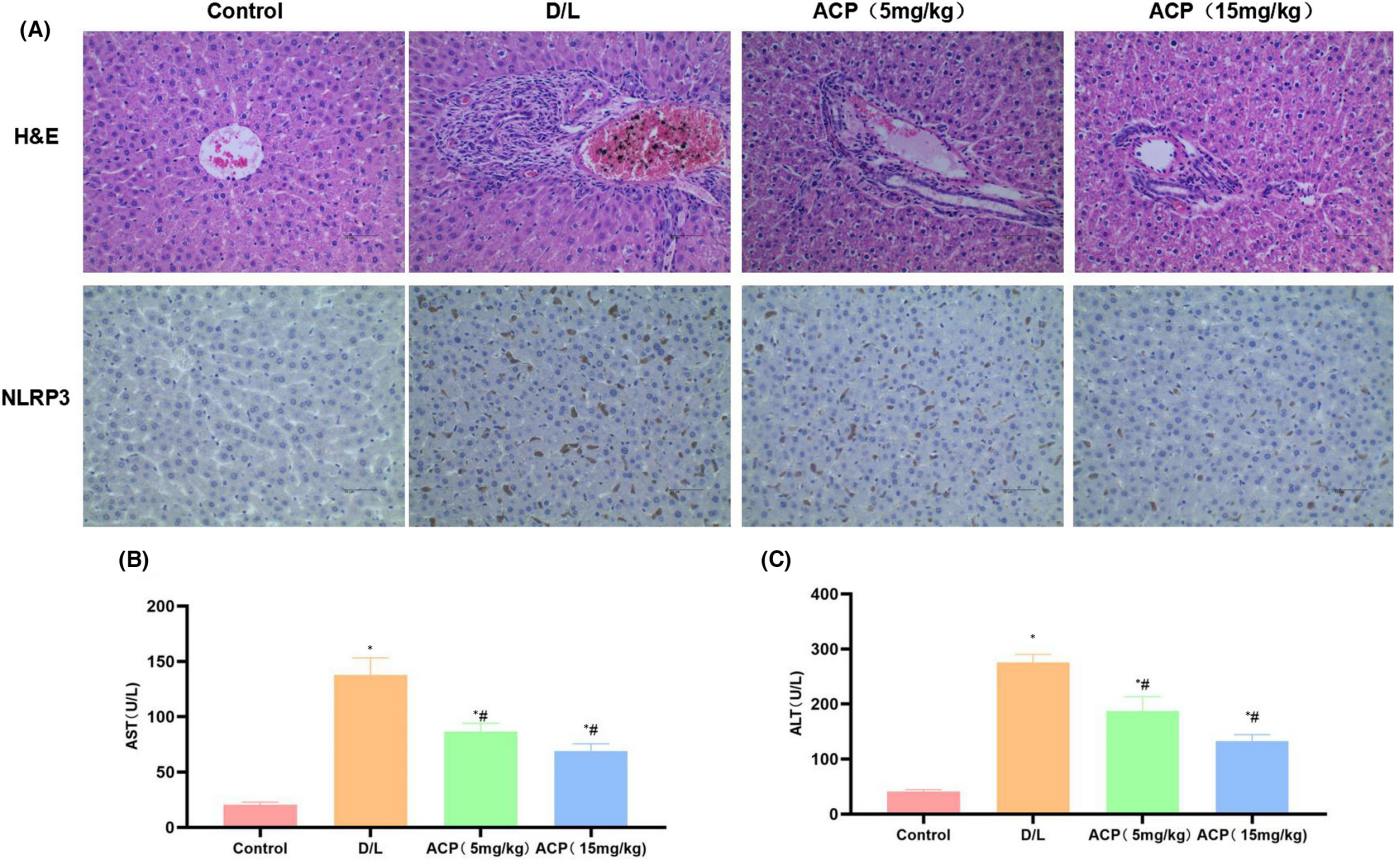
Effect of ACP on liver injury of mice. (A) H&E staining and immunohistochemical staining of mouse liver tissues (*n* = 5). NLRP3 expression was negative in Control group, while that in D/L group was up‐regulated, and ACP suppressed NLRP3 expression. H&E staining suggested that mouse liver tissues in D/L group experienced severe inflammation and oedema, with significant difference compared with Control group, while ACP improved inflammatory response and oedema. (B, C) ALT and AST expression in mice (*n* = 10). ALT and AST expression in peripheral blood of D/L mice was substantially up‐regulated, significantly higher than that in Control group. ACP intervention down‐regulated ALT and AST levels, and high‐dose ACP had superior effect to low‐dose ACP. ^*^ *p* < 0.05, compared with Control group; ^#^ *p* < 0.05, compared with D/L group

D/L promoted inflammatory response in mouse liver tissues and the activation of KCs, and up‐regulated NLRP3 expression. According to H&E staining, mice in D/L group had obvious pathological changes of liver tissues, inflammation and severe oedema, suggesting the presence of inflammatory injury in the liver tissues. After ACP intervention, NLRP3 expression was suppressed, the activation of KCs was inhibited, inflammatory response and level as well as tissue injury in mouse liver tissues were improved, proving that ACP obviously suppressed liver injury (Figure 6A). Moreover, AST and ALT detection results indicated that, mice in D/L group showed obvious liver injury, along with up‐regulated ALT and AST levels. ACP decreased ALT and AST levels, improved liver injury, and high‐dose ACP had superior effect compared with low‐dose ACP (Figure 6B, C).

**FIGURE 7:**
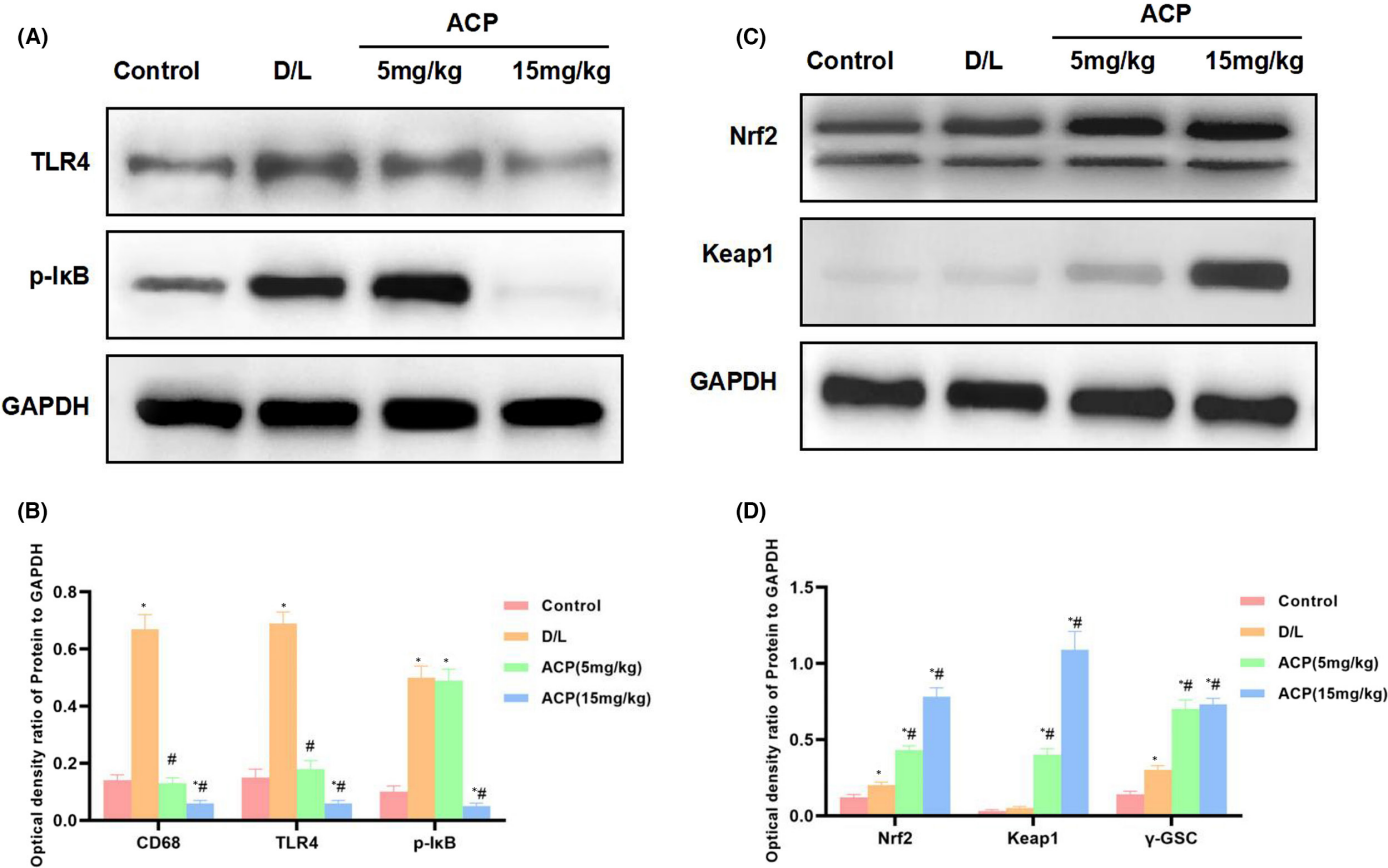
Effect on protein expression in mouse liver tissues (*n* = 3). TLR4 and I‐κB expression significantly increased in D/L group, higher than that in Control group. ACP intervention suppressed protein expression, inhibited TLR4 and I‐κB expression, activated the Nrf2 signal, and up‐regulated Nrf2 and keap1 protein levels. ^*^ *p* < 0.05, compared with Control group; ^#^ *p* < 0.05, compared with D/L group

According to protein detection results, ACP inhibited TLR4 and p‐IкB expression in liver tissues, activated Nrf2 signal, and increased Nrf2 and Keap1 expression, with significant differences compared with D/L group. Besides, high‐dose ACP had superior effect in comparison with low‐dose ACP (Figure 7).

## 4 DISCUSSION

Energy metabolism in organisms uses oxygen as the electron acceptor during the aerobic metabolism process, which inevitably produces reactive oxygen species (ROS).^13^ ROS is tightly associated with the regulation of some physiological activators and inflammatory immune processes, while excessive ROS is probably to induce OS.^14^ Research suggests that, the occurrence and development of liver diseases such as hepatitis, liver cirrhosis, fatty liver, liver cancer and alcoholic liver are closely related to free radical injury, and OS may be the common pathogenic mechanism of liver diseases.^15^, ^16^ Under pathological condition, the ability to scavenge ROS in the body decreases, and the excessive production of free radicals will lead to OS, inducing lipid peroxidation of polyunsaturated fatty acid in the biomembrane phospholipid, intracellular protein and enzyme denaturation, and DNA oxidative damage.^17^ Additionally, ROS serve as the important intracellular messengers to activate multiple signalling pathways, thereby indirectly inducing tissue and cell injury.^18^ Free radicals and their induced OS run through various liver diseases, which is one of the main mechanisms leading to liver diseases. NF‐κB is a kind of heterodimer constituted by p60 and p65 subunits, and it is the first transcription factor verified to directly respond to OS.^19^ In the complex cytokine network of inflammatory response induced by endotoxin or OS, the activation of NF‐κB may be a central step.^20^ Plenty of studies have confirmed that, reducing NF‐κB activity may alleviate ROS‐induced liver injury.^21^ Nrf2 is an important transcription factor that belongs to the leucine zipper family, which can regulate anti‐OS response and plays a pivotal role in the anti‐oxidation system.^22^ Under OS condition, Nrf2 is dissociated from its specific receptor Keap1 and is activated, which initiates the expression of phase II detoxifying enzyme and antioxidase genes regulated by antioxidant response element (ARE), and enhances resistance of cells to OS and electrophilic chemicals.^23^ In addition, nuclear transcription factor AP‐1 is also the hotspot in current OS research, and its activation plays an important role in OS and inflammatory cascade reaction of liver diseases, which is promising to become a new approach to treat diverse liver diseases.^24^

Antrodia Camphorata (AC) is a kind of Polypores family fungus unique to Taiwan, and it is currently artificially cultured in Mainland China. Amongst the active ingredients of AC, polysaccharide and triterpene are two natural products with rich contents. According to the literature reports, AC generates favourable therapeutic effect on liver diseases such as hepatitis, liver injury and liver cancer, while its active ingredients and exact mechanism of action remain unclear. This study treated KCs as the objects and discovered that ACP suppressed the LPS‐induced KC cell activation, and such action was related to ROS suppression. ROS is one of the important induction factors of TLR4 signals,^25^ which can activate TLR4 receptor through OS injury, thus regulating the activation of the downstream NF‐κB.^26^ After KCs are activated, CD68 expression is up‐regulated. This is because that under inflammatory condition, KCs are inclined to M1 polarization of macrophages, a kind of pro‐inflammatory effect, while CD68 is an important protein of M1 polarization.^27^ The most significant effect of LPS is to promote the release of inflammatory factors in KCs, while ACP significantly down‐regulates the expression of inflammatory factors. More importantly, ACP promotes SOD expression, which is a kind of anti‐OS effect. To further explore the mechanism of ACP, we used the ROS inhibitor NAC in combination with ACP, and discovered the synergistic effect of the two, which suppressed KC activation by suppressing ROS. Nrf2 is an important antioxidant signal pathway. It could be found that ACP exerted the similar effect to Nrf2 agonist BCI, and activated Nrf2 expression and nuclear translocation. Therefore, we speculate that the effect of ACP against ROS is related to the activation of Nrf2 signal.

In the animal model research, D‐GalN/LPS induced the occurrence of liver injury, and liver tissues experienced obvious inflammatory response and oedema. Most importantly, D‐GalN/LPS induced the high expression of ROS and the activation of KCs. On this basis, we applied ACP intervention and discovered that ACP suppressed the expression of CD68 and the inflammatory factor TNF‐α, inhibited inflammatory response and the activation of KCs, and down‐regulated the ALT and AST levels in peripheral blood. Apparently, ACP had excellent liver protection effect. Similarly, it was also found from ROS detection that, ACP decreased ROS expression to further suppress the activation of TLR4‐NF‐κB signal and activate Nrf2 signal, consistent with cellular experimental results.

## 5 CONCLUSION

This study discovers that ACP activates Nrf2 signal to suppress ROS expression, further regulates the ROS‐induced TLR4‐NF‐κB signal, and exerts an important liver protection effect in liver injury. Moreover, this study provides pharmacodynamic material basis for the liver protection of AC and further reveals the role of polysaccharide in liver injury.

## CONFLICT OF INTEREST

No competing interests.

## AUTHOR CONTRIBUTIONS

**Yi Yang:** Conceptualization (equal); Data curation (equal). **Chenyang Han:** Formal analysis (equal); Funding acquisition (equal). **Yongjia Sheng:** Data curation (equal); Resources (equal); Software (equal). **Jin Wang:** Investigation (equal); Methodology (equal); Supervision (equal). **Wenyan Li:** Investigation (equal); Resources (equal); Supervision (equal); Writing – review & editing (equal). **Xiaohong Zhou:** Supervision (equal); Validation (equal); Visualization (equal). **Shuiliang Ruan:** Funding acquisition (equal); Visualization (equal); Writing – review & editing (equal).

## CONSENT FOR PUBLICATION

All authors approval published the article.

## Data Availability

The data and material were availability.

## References

[1] Ali M , Obeid AK . Evaluation of the effect of aqueous extract of lepidium sativum seed on the adverse effects of rat liver injury induced by sodium nitrite. Trop J Pharm Res. 2020;12(4):975‐2366.

[2] Wensink D , Wagenmakers MAEM , Wilson JHP , Langendonk JG . Letter to the editor: Diagnosis of erythropoietic protoporphyria with severe liver injury‐a case report. World J Gastroenterol. 2019;30:4292‐4293. 10.3748/wjg.v25.i30.4292 PMC6700695 31435180

[3] Ganesan M , Poluektova LY , Kharbanda KK , et al. Human immunodeficiency virus and hepatotropic viruses comorbidities as the inducers of liver injury progression. World J Gastroenterol. 2019;25(4):398‐410. 30700937 10.3748/wjg.v25.i4.398 PMC6350175

[4] Ning HQ , Li YQ , Tian QW , et al. The apoptosis of Staphylococcus aureus induced by glycinin basic peptide through ROS oxidative stress response. Lebensmittel Wissenschaft Und Technologie. 2019;99:62‐68.

[5] Barbouti A , Vasileiou PVS , Evangelou K , et al. Implications of Oxidative Stress and Cellular Senescence in Age‐Related Thymus Involution. Oxidat Med Cell Long. 2020;2020(6):1‐14. 10.1155/2020/7986071 PMC7025075 32089780

[6] Matsuzawa A , Saegusa K , Noguchi T , et al. ROS‐dependent activation of the TRAF6‐ASK1‐p38 pathway is selectively required for TLR4‐mediated innate immunity. Nat Immunol. 2005;6(6):587‐592. 15864310 10.1038/ni1200

[7] Nishanth RP , Jyotsna RG , Schlager JJ , et al. Inflammatory responses of RAW 264.7 macrophages upon exposure to nanoparticles: role of ROS‐NFκB signaling pathway. Nanotoxicology. 2011;5(4):502‐516. 21417802 10.3109/17435390.2010.541604

[8] Bian X , Liu X , Liu J , et al. Hepatoprotective effect of chiisanoside from *Acanthopanax sessiliflorus* against LPS/D‐GalN‐induced acute liver injury by inhibiting NF‐κB and activating Nrf2/HO‐1 signaling pathways. J Sci Food Agric. 2019;99(7):3283‐3290. 30552777 10.1002/jsfa.9541

[9] Je J , Kim H , Park EJ , et al. Fermentation of sprouted ginseng (*Panax ginseng*) increases flavonoid and phenolic contents to attenuate alcoholic hangover and acute liver injury in mice. Am J Chinese Med. 2020;1:1‐16. 10.1142/S0192415X21500075 33371811

[10] Deng JS , Huang SS , Lin TH , et al. Analgesic and anti‐inflammatory bioactivities of eburicoic acid and Dehydroeburicoic acid isolated from *Antrodia camphorata* on the inflammatory mediator expression in mice. J Agri Food Chem. 2013;61(21):5064‐5071. 10.1021/jf303820k 23495748

[11] Tzong‐Huei L , Chien‐Chih C , Jih‐Jung C , et al. New and cytotoxic components from *Antrodia camphorata* . Molecules. 2014;19(12):21378‐21385. 25532837 10.3390/molecules191221378 PMC6271605

[12] Elliott EI , Sutterwala FS . Initiation and perpetuation of NLRP3 inflammasome activation and assembly. Immunol Rev. 2015;265(1):35‐52. 25879282 10.1111/imr.12286 PMC4400874

[13] Li M , Wang S , Li X , et al. Diallyl sulfide treatment protects against acetaminophen‐/carbon tetrachloride‐induced acute liver injury by inhibiting oxidative stress, inflammation and apoptosis in mice. Toxicol Res. 2019;8(1):67‐76. 10.1039/c8tx00185e PMC6334500 30713662

[14] Sun JC , Du JJ , Li XQ , et al. Depletion of β‐arrestin 2 protects against CCl4‐induced liver injury in mice ‐ ScienceDirect. Biochem Biophys Res Comm. 2020;522(2):485‐491. 31780259 10.1016/j.bbrc.2019.11.093

[15] Tanimizu N , Ichinohe N , Suzuki H , et al. Prolonged oxidative stress and delayed tissue repair exacerbate acetaminophen‐induced liver injury in aged mice. Aging. 2020;12(19):18907‐18927. 33001859 10.18632/aging.103973 PMC7732315

[16] Wu CT , Deng JS , Huang WC , et al. Salvianolic Acid C against Acetaminophen‐Induced Acute Liver Injury by Attenuating Inflammation, Oxidative Stress, and Apoptosis through Inhibition of the Keap1/Nrf2/HO‐1 Signaling. Oxidat Med Cell Long. 2019;2019:1‐13. 10.1155/2019/9056845 PMC6535820 31214283

[17] Kalantar M , Kalantari H , Goudarzi M , Khorsandi L , Bakhit S , Kalantar H . Crocin ameliorates methotrexate‐induced liver injury via inhibition of oxidative stress and inflammation in rats. Pharmacol Rep. 2019;71(4):746‐752. 31220735 10.1016/j.pharep.2019.04.004

[18] Shu G , Qiu Y , Hao J , et al. Nuciferine alleviates acute alcohol‐induced liver injury in mice: Roles of suppressing hepatic oxidative stress and inflammation via modulating miR‐144/Nrf2/HO‐1 cascade. J Funct Foods. 2019;58:105‐113.

[19] Dias AS , Porawski M , Alonso M , et al. Quercetin decreases oxidative stress, NF‐κB activation, and iNOS overexpression in liver of streptozotocin‐induced diabetic rats. J Nutr. 2005;135(10):2299‐2304. 16177186 10.1093/jn/135.10.2299

[20] Rajakumar D , Alexander M , Oommen A . Oxidative stress, NF‐κB and the ubiquitin proteasomal pathway in the pathology of calpainopathy. Neurochem Res. 2013;38(10):2009‐2018. 23846623 10.1007/s11064-013-1107-z

[21] Jo HS , Yeo EJ , Shin MJ , et al. Tat‐DJ‐1 enhances cell survival by inhibition of oxidative stress, NF‐κB and MAPK activation in HepG2 cells. Biotech Lett. 2017;39(4):1‐11. 10.1007/s10529-017-2286-5 28074428

[22] Chan K , Han XD , Kan YW . An important function of Nrf2 in combating oxidative stress: detoxification of acetaminophen. Proc Natl Acad Sci. 2001;98(8):4611‐4616. 11287661 10.1073/pnas.081082098 PMC31882

[23] Hybertson BM , Gao B , Bose SK , et al. Oxidative stress in health and disease: the therapeutic potential of Nrf2 activation. Mol Aspects Med. 2011;32(4–6):234‐246. 22020111 10.1016/j.mam.2011.10.006

[24] Jia G , Law GL , Wong KL , et al. Exercise stimulates transcription factors (Nrf2 & NFkB), increases antioxidant defenses, decreases oxidative stress, and restores renal dopamine d1 receptor function in aging. FASEB J. 2000;22(2):251‐263.

[25] Yang ZC , Wang KS , Wu Y , et al. Asymmetric dimethylarginine impairs glucose utilization via ROS/TLR4 pathway in adipocytes: an effect prevented by vitamin E. Cell Physiol Biochem. 2009;24(1–2):115‐124. 19590199 10.1159/000227819

[26] Duan H , Qu L , Shou C . Mycoplasma hyorhinis induces epithelial‐mesenchymal transition in gastric cancer cell MGC803 via TLR4‐NF‐κB signaling. Cancer Lett. 2014;354(2):447‐454. 25149064 10.1016/j.canlet.2014.08.018

[27] Luo W , Xu Q , Wang Q , et al. Effect of modulation of PPAR‐γ activity on Kupffer cells M1/M2 polarization in the development of non‐alcoholic fatty liver disease. Sci Rep. 2017;7(1):44612. 28300213 10.1038/srep44612 PMC5353732

